# Attitudes about Dementia in Different Stages of Adulthood

**DOI:** 10.1093/geroni/igab046.2308

**Published:** 2021-12-17

**Authors:** Melissa O'Connor, Megan Pedersen, Susan McFadden

**Affiliations:** 1 North Dakota State University, Moorhead, Minnesota, United States; 2 North Dakota State University, Fargo, North Dakota, United States; 3 University of Wisconsin Oshkosh, Oshkosh, Wisconsin, United States

## Abstract

Research on attitudes toward dementia has often focused on younger and older adults; few studies have also included the age groups of established and middle adulthood. The current study utilized data from community-dwelling adults aged 18-95 (n=567) residing in two Midwestern states. Participants were divided into four age groups: emerging/young adulthood (ages 18-29), established adulthood (ages 30-45), middle adulthood (ages 46-64), and older adulthood (age 65+). ANOVA models were used to examine age group differences on the following outcomes: factual knowledge about dementia (total score on 14 true-false questions); attitudes toward dementia (total score on the 20-item Dementia Attitudes Scale); and a single item, “I am afraid of losing my memory” (rated on a 5-point scale). The effect of age group was significant in all models (p<0.01 for all). Emerging/young adults had significantly more knowledge about dementia, but less positive attitudes toward dementia, relative to established, middle-aged, and older adults. Attitudes and knowledge did not differ between established, middle-aged, and older adults. By contrast, older adults reported significantly more fear of memory loss than emerging/young, established, and middle-aged adults; fear did not differ between the latter three age groups. There were no significant interactions between age group and sex in any of the models. Implications of these findings are discussed.

